# Intracavernous administration of bone marrow mononuclear cells: a new method of treating erectile dysfunction?

**DOI:** 10.1186/1479-5876-11-139

**Published:** 2013-06-09

**Authors:** Thomas E Ichim, Timothy Warbington, Octav Cristea, Joseph L Chin, Amit N Patel

**Affiliations:** 1 Institute for Molecular Medicine, Huntington Beach, CA, USA; 2 Creative Medical Health Inc, 2007 W Peoria Avenue, Phoenix AZ 85029, USA; 3 University of Western Ontario, London, Canada; 4 University of Utah, Salt Lake City, UT, USA

## Abstract

While PDE5 inhibitors have revolutionized treatment of ED, approximately 30% of patients are non-responsive. A significant cause of this is vascular and smooth muscle dysfunction, as well as nerve atrophy. Autologous administration of bone marrow mononuclear cells (BMMC) has been performed in over 2000 cardiac patients without adverse effects, for stimulation of angiogenesis/regeneration. Despite its ease of access, and dependence on effective vasculature for function, comparatively little has been perform in terms of BMMC therapy for ED. Here we outline the rationale for use of autologous BMMC in patients with ED, as well as provide early safety data on the first use of this procedure clinically.

## Introduction

Erectile responses require a coordinated increase in arterial inflow, which originates from the pudendal arteries, relaxation of the corporal smooth muscle, and inhibition of venous outflow [1,2]. Key to this response is production of nitric oxide (NO) from endothelial cells and nonadrenergic noncholinergic (NANC) postganglionic parasympathetic neurons, as well as responsiveness to this. NO binds to, and activates, the enzyme guanylate cyclase, which in turn catalyzes the generation of cGMP from GTP. As a result, cGMP induces a cascade of signals in the smooth muscle cells resulting in relaxation [3]. Breakdown of cGMP in the cavernosal tissue is mediated by PDE-5. Increasing the duration of NO signaling by preventing cGMP breakdown is the main mechanism of action for the successful PDE-5 inhibitor class of drugs which currently are used as first-line treatment of ED [4]. Interestingly, recent studies have shown that these drugs have other beneficial effects such as stimulation of bone marrow endothelial progenitor cell function [5-9], inhibition of smooth muscle cell apoptosis [10,11], preservation/restoration of function in post-prostatectomy settings [12,13] and activation of mesolimbic dopaminergic neurons in the CNS to promote sexual behavior [14].

Unfortunately, a significant number of patients are resistant to effects of PDE5 inhibitors [15]. Major factors associated with this include atherosclerosis, nerve damage and smooth muscle atrophy [16]. Several approaches have demonstrated some promise in the improvement of responsiveness to PDE5 inhibitors including propionyl-L-carnitine [17,18], intracavernous PGE1 [19], and testosterone gel [20,21]. However these studies are early and do not address the underlying biological cause in many of the situations of ED. Since the majority of ED cases appear to be a manifestation of systemic atherosclerotic disease [22,23], and various forms of stem cell therapy have shown some efficacy in other manifestation of atherosclerotic disease [24-35], the possibility of applying such regenerative approaches to ED has been considered by investigators in animal models [36-48].

## Circulating endothelial progenitor cell dysfunction in ED

Atherosclerosis and endothelial dysfunction of the penile microvasculature is one of the major causes of ED. This is particularly relevant since the penile arteries have the smallest diameter of the vascular network and thus are the most sensitive to these changes [49]. Therefore in order to develop means to treat ED, it is important to understand how the vasculature self-renews itself. The bone marrow serves as a continuous supply of circulating endothelial progenitor cells (EPC) for the systemic vasculature. The concept of endothelial renewal by circulating cells was described by Asahara et al who demonstrated that cells expressing VEGFR-2 and CD34 were capable of incorporating into sites of active angiogenesis induced by wire injury or ischemia. The authors of the study found comparable cells in the human system [50]. Subsequent studies have shown that several subtypes of circulating EPC exist, with some capable of giving rise to early colonies of endothelial cells in vitro and others giving rise to late colonies [51]. In general, the majority of studies assessing EPC function in humans detect the cells using a combination of the CD34, AC133, and VEGFR-2 markers, although both the early and late outgrowth populations of cells are present in this phenotypic subset [52].

Increases in circulating EPC have been described in studies of acute inflammation such as myocardial infarction and stroke [53-57]. It has been demonstrated that tissue injury causes site-specific upregulation of chemotactic factors such as stromal derived factor (SDF)-1, which in turn mobilize EPC from bone marrow compartments into the site of injury to participate in formation of new blood vessels [58]. Supporting this, positive correlations have been found between post-stroke increase in circulating EPC and better prognosis [59]. Conversely, basal low levels of EPC predict cardiovascular events [60]. Numerous studies have demonstrated in animal models that administration of exogenous EPC increases vascular repair. This has been shown using in vitro generated EPC, or bone marrow as a source of EPC in myocardial infarct [61,62], stroke [63], lung injury [64-66], liver failure [67-69], and endothelial injury atherosclerotic models [70,71]. Furthermore, administration of growth factors that stimulate mobilization of bone marrow stem cells and EPC have demonstrated therapeutic benefit in animal models of ischemic disease [72,73] as well as endothelial damage [74]. Clinical trials administering EPC or bone marrow as a source of EPC for cardiovascular conditions [28,75-77], have demonstrated some therapeutic benefit, although work is ongoing. Indeed various other factors may be needed to augment efficacy. For example, it was recently discovered that testosterone levels correlate with ability of EPC to function [78,79]. Specifically, castrated mice possess marked deficiencies in ability to undergo spontaneous angiogenesis in responses to hindlimb ischemia [80]. In the cardiac studies testosterone levels were not tested, and it is believed that a significant segment of the older population has a deficiency in testosterone [81].

In states of chronic inflammation, EPC activity is decreased. Specifically, conditions such as diabetes [82-86], hypercholesteremia [87-92], obesity [93,94] and cardiovascular disease [95,96] all are associated with decreased circulating EPC compared to controls. Interestingly, in volunteers that do not suffer from cardiovascular disease but have cardiovascular disease risk factors as assessed by the Framingham risk factor score, a negative correlation is found between cardiovascular risk and EPC function. Unhealthy lifestyle such as smoking also decreases EPC. In a study by Kondo et al undetectable levels of EPC were found when colony formation was assessed, and significantly reduced levels of cells possessing EPC phenotype were found in smokers compared to healthy controls [97]. Smoking cessation for 4 weeks was capable of increasing EPC numbers, whereas when subjects restarted smoking after the 4 weeks, EPC levels dropped again.

Since ED appears to be one of the early manifestations of systemic cardiovascular disease, it is not surprising that ED patients possess a deficiency in circulating EPC. In a study by Baumhäkel et al, numbers of of CD34(+)/KDR(+) and CD133(+) cells were assessed in 119 coronary artery disease patients. Prevalence of ED, as assessed by the KEED questionnaire was 59.7% in this population. Low levels of CD133 cells were identified as an independent risk factor for ED when adjustments for age, diabetes, hypertension, BMI, smoking, LVEF, use of statins and lower urinary tract symptoms, and prior coronary intervention [98]. A subsequent study by Esposito et al in 60 otherwise healthy overweight men of which 30 suffered from ED and 30 did not, revealed a significant direct correlation between circulating CD34(+)KDR(+) cells and erectile function as assessed by the International Index of Erectile Function (IIEF) questionnaire [99]. Foresta et al utilized high resolution echo color doppler to quantify penile atherosclerosis associated with ED by measuring the intima media thickness [IMT] in the penile vasculature before and after intracavernous alprostadil injection. Twenty patients with ED and 15 controls were recruited for the study. A progressive reduction of circulating EPC with the severity of cavernous artery atherosclerosis was found [100].

Given the low number of EPC in heart failure patients, it may be reasonable to believe that there is a diminished regenerative capacity of the endothelium in the cavernousum. Thus administration of cells possessing EPC function may be useful.

## Angiogenic cytokines and ED

Cytokines play a critical role in coordinating the process of angiogenesis and vascular renewal by EPC. SDF-1 is a fundamental factor in stimulation of angiogenesis, which functions to attract EPC to areas of injury [101]. Conditions of reduced blood flow or hypoxia induce activation of HIF-1 alpha, which in turn stimulate expression of SDF-1 [102]. SDF-1 administration has been demonstrated to augment activity of endogenous EPC and promote neovascularization in the cardiac setting [103]. On the other hand, various cytokines within the corpus cavernosum are needed for EPC to integrate and form new blood vessels. In ED it has been found that levels of angiogenic cytokines such as VEGF and FGF are reduced. In a rat model of ED induced by hypercholesteremia, a negative correlation between VEGF, angiopoietin-1, and angiopoietin-2 and erectile function was observed at the gene and protein level in cavernous tissue [104]. In a rabbit hypercholesteremia model, reduction in FGF-2 is observed in the cavernous tissue, and administration of this protein intracavernously results in a VEGF dependent restoration in function [105]. A possible functional correlation between decreased VEGF expression in the corpus cavernosum and ED is suggested in a study which showed rabbits fed a high cholesterol diet had a decrease in VEGF expression before onset of ED [106]. While in general, it appears that the process of aging decreases VEGF expression in the penile tissue [107,108], other factors associated with ED such as hyperglycemia [108], androgen deficiency [109], and chronic ischemia [110] appear to further cause decrease in VEGF expression.

Given that cellular therapy evolved after cytokine/gene therapy, numerous studies have been conducted assessing efficacy of administration of cytokines in models of ED. The rationale being that not only would transfer of agents such as VEGF augment neoangiogenesis and endothelial rejuvenation, but that they would also prevent apoptosis of endothelial cells as well as neurons in the cavernosum. Byrne et al. reported that a single intracavernous injection of VEGF protein or systemic injection was capable of restoring to normal in vitro smooth muscle relaxation in cholesterol-fed rabbits. Smooth muscle relaxation induced by both acetylcholine, which is endothelium dependent and sodium nitroprusside, which is NO mediated, was restored. Interestingly the authors found an increase in smooth muscle content of the cavernosum in animals that received intracavernous injection of VEGF but not systemic administration [111]. A previous preliminary study by the same group reported similar effects of VEGF when administered weekly for 4 weeks [112]. Subsequent studies have demonstrated that viral vector administration of VEGF is capable of restoring erectile function in testosterone deficient models of ED [113]. The effects of VEGF appear to be therapeutic in a variety of models of ED, for example Park et al demonstrated improvement in the aged rat model [114], Dall’Era et al demonstrated effects in the diabetes model [115], and Hsieh et al demonstrated efficacy in a crush-injury model [116].

Microarray analysis of rats with penile hypocirculation induced by pudendal artery ligation revealed that VEGF administration into the corpus cavernosum is associated with upregulation of eNOS and iNOS genes at 6 and 24 hours post administration [117]. Additional mechanisms of VEGF on ED include upregulation of eNOS function by phosphorylation on a specific serine residue [118]. Other mechanisms of VEGF on erectile function include stimulation of anti-apoptotic genes such as bcl-2 in the cavernosum [119], and modulation of the insulin-like growth factor system and sex hormone receptors [120].

Other angiogenic growth factors have been demonstrated to increase erectile function in animal models. For example, FGF-2, a heparin-binding growth factor has been demonstrated to increase smooth muscle content and prevent histological changes associated with ED in a hypercholesterolemia rabbit model subsequent to systemic administration [121]. Interestingly, therapeutic benefit was associated with augmentation of VEGF expression. Subsequent studies have demonstrated that local FGF-2 administration is capable of augmenting vasoreactivity of the corpus cavernosum in a similar model system [122]. IGF-1 is known to act as an anti-apoptotic molecule in several systems and stimulates angiogenesis, in part through induction of VEGF and VEGF receptor expression [123]. Suppression of IGF-1 production at a local level is found in uremia induced ED in animal models [124], and reduction at a systemic level is associated with aging and obesity [125,126]. The possibility that IGF-1 may be therapeutic in ED was suggested by studies in which regeneration of penile nerves was associated with upregulation of IGF-1 in a cavernous neurotomy model [127]. Administration of IGF-1 via adenoviral delivery into the penis was demonstrated to improve erectile function and smooth muscle mass in a streptozotocin-induced model of diabetes associated ED [128]. Furthermore, these date were confirmed in an age-associated rat model of ED, in which it was demonstrated that the effects of IGF-1 were mediated at least in part by stimulation of eNOS synthesis as well as augmented concentrations of cGMP [129].

Thus while it appears that VEGF, FGF-2 and IGF-1 are potential candidates for stimulation of cavernosum regeneration/rejuvenation therapy, trials using these agents in other cardiovascular conditions have yielded poor results [130]. Furthermore, although gene therapy into the corpus cavernosum has been demonstrated to possess promising safety data in early human trials [131], little work to our knowledge is being performed in this space. Cellular therapy possesses the potential advantages of: a) production of a regulated “symphony of therapeutic cytokines” based on the need of the local environment; b) relatively lower risk level, especially in autologous, non-expanded settings; and c) the ability of the cells to differentiate into effector cells. Therefore we will review previous work performed on cell therapy for ED.

## Previous cellular therapy approaches to ED

Bone marrow stem cells have been used for over 4 decades in the area of hematopoietic stem cell transplantation. Stimulation of angiogenesis using this cell population has been performed in animal models of ischemia, as well as in clinical trials [132]. Kendirci et al used bone marrow cells that were isolated for expression of the p75 nerve growth factor receptor using magnetic activated cell sorting. They chose this population based on possible enhancement of neurogenic potential. Intracavernous administration of these cells into a rat bilateral cavernous nerve crush injury model was performed. At 4 week follow up, improvement in erectile function as assessed by mean intracavernous-to-mean arterial pressure ratio and total intracavernous pressure was assessed. Significant improvements were observed in animals receiving the p75 selected cells as compared to those receiving an equal concentration of bone marrow derived multipotent stromal cells, fibroblasts, or saline. Significantly higher levels of FGF-2 were found in the cavernosum of animals receiving the p75 selected cells [38]. To our knowledge this is the only animal experiments that utilized bone marrow derived cells without expansion in vitro.

The possibility of using mesenchymal stem cells in the treatment of ED is enticing not only because these cells are known to secrete various growth factors that are beneficial in ED such as IGF-1 [133-135], VEGF [136], and FGF-2 [137], but also because of their anti-inflammatory activities [138], as well as possibility of differentiating into tissue relevant to the penile architecture [139]. To assess whether bone marrow derived MSC had a therapeutic effect on diabetes induced ED, Qiu et al performed intracavernous administration of these cells. Four weeks after administration, the ratio of intracavernous pressure and mean arterial pressure (ICP/MAP ratio), as well as smooth muscle and endothelial cell compartment was significantly upregulated compared to controls. Cell tracking experiments revealed that the MSC were retained for at least 4 weeks post injection and showed expression of endothelial and smooth muscle cell markers, suggesting the possibility of transdifferentiation [37]. A subsequent study examined long term effects of MSC administration via the intracavernous route in aged rats. The study found that the mean cavernous cGMP levels after 3 and 4 months of MSCs transplantation were increased compared with those after 3 or 4 weeks, which were in turn higher than controls. Cavernous tissue ICP measurement showed significant increase in MSCs transplanted groups compared with the controls, which was more significant in the long-term follow up [40]. This suggests that some of the therapeutic effects of regenerative therapy may be observed in a more delayed setting as opposed to some of the previously mentioned gene therapy approaches. Similar therapeutic effects were observed with muscle derived MSC in the aged rat model, however long term follow-up was not performed [44]. Given that MSC may be used clinically in an allogeneic model, a xenogeneic model of human MSC into immune competent rats was performed. Administration of an immortalized human MSC clone into the cavernosum of Sprague Dawley rats resulted in differentiation into endothelial and smooth muscle cells [46]. Non-invasive imaging studies by the same group reported that human MSC may be found up to 12 weeks post injection in the cavernosum of rabbits and rats [45]. In order to augment therapeutic efficacy of MSC, genes for VEGF and eNOS were transfected into MSC for treatment of diabetes and age-associated ED, respectively. In both cases significant improvements in therapeutic efficacy were observed when gene transfected MSC were used in comparison to MSC alone [36,47].

Adipose tissue derived stromal vascular fraction (SVF) cells represent a potent source of EPC, MSC and hematopoietic stem cells that has been used in clinical pilot trials and is part of veterinary medical practice in the USA [140]. The MSC component from SVF is postulated to possess some unique advantages to bone marrow MSC, such as augmented angiogenic activity, however this is controversial [43]. Several studies have used adipose derived mesenchymal stem cells that were in vitro expanded for the treatment of ED in the cavernosal nerve injury model [39], the hyperlipidemia model [41], and the streptozotocin induced diabetes model of ED [42]. Unfortunately it is still not clear which stem cell source is better since back to back experiments have not been performed. Given the potent angiogenic characteristics of the endometrial-derived MSC, termed endometrial regenerative cells (ERC) [141], it may be relevant to assess therapeutic effect of these cells in models of ED.

Clinical use of stem cells in treatment of ED has been reported by Bahk et al from Korea who treated 7 patients with diabetes associated ED which was unresponsive to medication for at least 6 months with an average of 1.5 × 10(7) cord blood mononuclear cells injected intracavernously. Three additional patients with similar characteristics were used as controls [142]. No treatment associated abnormalities were reported despite the allogeneic nature of the cells in absence of immune suppression. One month after treatment, morning erections were regained in 3 participants. By the third month post treatment 6 of the 7 patients had regained morning erections. In all patients rigidity increased as the result of cord blood administration, but was not sufficient for penetration. When the patients were administered PDE5 inhibitor before coitus, 2 achieved penetration and experienced orgasm, and maintained for more than 6 months; however, 1 participant could not achieved penetration at ninth month. Interestingly, an increase in sexual desire was reported in 6 of the 7 patients. No improvements were observed in any of the 3 control patients.

Overall these studies support: a) the rational for use of various adult stem cells in the treatment of ED, and b) the preliminary human feasibility.

## Bone marrow mononuclear cells

Bone marrow mononuclear cells have been used as a stem cell source for over 40 years in the field of hematological transplantation [143]. Non-hematopoietic uses of bone marrow mononuclear cells have historically included transplantation for post infarct recovery of the heart. The rationale behind this is that bone marrow cells contain angiogenic cells [144], which support production of new blood vessels and accelerate healing of the infracted scar. Chimeric studies demonstrated a critical role of the c-kit receptor in that bone marrow from c-kit mutant mice was not able to reverse pathological remodeling and inhibit infarct size, post infarct [145]. Additionally, it is believed that bone marrow derived cells are capable of directly differentiating into myocardial tissue [146]. This rationale stimulated the first report of bone marrow administration for treatment of post infarct cardiac damage.

In 2001 Strauer et al [147], reported a case report of a 46-year-old man who suffered a transmural infarction as a result of an occluded anterior descending branch of the left coronary artery. Six days after the infarct and subsequent to angioplasty and stent placement, the patient was administered 1-2 × 10(7) bone marrow mononuclear cells via a percutaneous transluminal catheter placed in the infarct-related artery. At 10 weeks after the stem cell transplantation the infarct area was diminished from 24.6% to 15.7% of left ventricular circumference, while ejection fraction, cardiac index and stroke volume were increased by 20-30%. Exercise-induced end diastolic volume was decreased by 30% and a similar decrease in mean pulmonary capillary pressure was observed. A subsequent study of 9 post infarct patients receiving autologous bone marrow into infarct related artery revealed improvements in ejection fraction and diminished improved regional wall motion in the infarct zone at 4 month follow-up. Additionally at the same time point a reduction in end-systolic left ventricular volumes. In the historical control group no significant change in ejection fraction, nor end-systolic volumes was observed [148]. Although larger double blind trials have reported mixed results [149-151], the overall consensus is that bone marrow administration post infarct induces a mild benefit in terms of ejection fraction and reduction in pathological remodeling [152,153].

In addition to post-infarct healing, bone marrow mononuclear cells have been extensively used for the direct stimulation of angiogenesis. In the cardiac arena, one of the first stem cell uses was reported by Hamano et al in 2001 [154], who used autologous bone marrow implantation into the ischemic area of patients with ischemia heart disease undergoing coronary artery bypass surgery. At 1 year follow-up 3 of the 5 patients treated reported objective functional improvement with angiogenesis visualized at the points of injection by imaging [154]. Subsequent studies have been conducted demonstrating benefit of direct intramyocardial injections of bone marrow mononuclear cells. Beeres et al [155]. reported improved exercise capacity, ejection fraction, and quality of life at 3 and 6 month timepoints after autologous bone marrow therapy in severe angina. A 50-patient double blinded study of myocardial ischemia patients who were non-responsive to medical intervention and ineligible for coronary revascularization demonstrated a statistically significant improvement in cardiac perfusion using autologous bone marrow mononuclear cells implanted intramyocardially [156].

Critical limb ischemia (CLI) is a severe form of peripheral artery disease whose only treatment is percutaneous or surgical revascularization for patients who have favorable anatomy. Patients who do not, usually require amputation. Formation of collateral blood vessels surrounding the area of occlusion is a well documented phenomenon in patients with CLI (reviewed in ref [157]) and is believed to be caused by circulating stem/progenitor cells that cause localized angiogenesis. Indeed because of these previous observations, investigators have questioned whether the process of endogenous angiogenesis could be augmented by intramuscular implantation of autologous bone marrow mononuclear cells into the ischemic limb. The first clinical trial using this procedure was reported by Tateishi-Yuyama et al. who reported a statistically significant increase in perfusion, walking distance, and oxygenation of ischemic legs as compared to baseline in one group, and in another study group as compared to injection of peripheral blood mononuclear cells [158]. Subsequent groups have repeated the finding that autologous bone marrow mononuclear cells have a therapeutic effect on angiogenesis in the ischemic leg. Nizankowski et al reported reduction of pain and improved perfusion in 10 patients with Fountaine IV class CLI [159]. A similar open label study in 12 CLI patients demonstrated improvements in resting ankle-brachial pressure index (ABI), arterial oxygen saturation (SaO(2)), pain-free walking time and rest pain scale evaluation [160]. A larger, 51 patient study, demonstrated improvement in a mean Rutherford category of 4.9 at baseline to 3.3 at 6 months, as well as reduction in analgesics consumption by 62%. Perfusion was increased as detected by ankle brachial index and transcutaneous oxygen. Furthermore, total walking distance improved in nonamputees from zero to 40 m [161].

Thus the process of systemic or local administration of autologous bone marrow mononuclear cells has been shown to have therapeutic effects in ischemia associated heart failure and limb dysfunction. In other conditions such as liver failure [162] and stroke [163], studies have shown this source of cells mediates therapeutic effects, possibly in part by stimulation of angiogenesis [164].

Given that bone marrow mononuclear cells can be extracted and concentrated in FDA-approved closed system devices, and are already being used under the practice of medicine for a variety of indications, we sought to explore the safety and feasibility of intracavernous administration of these cells in a patient suffering from erectile dysfunction.

## Case report

A 35 year old patient presented to us with a history of erectile dysfunction unresponsive to oral PDE5 inhibitors. The patient was a smoker and had a history of hypercholesteremia, marginal effects from intracorporal PGE1 (Caverject) administration, 2 years ago, but as of 6 months, the treatment had no effect. Psychogenic ED was discounted based on 2 independent nocturnal penile tumescence (NPT) tests, which revealed abnormal findings. Normal was defined as having at least 1 episode of nocturnal erection of at least 10 minutes duration with a 2-cm increase in tumescence of the tip and 3-cm increase in tumescence of the base, together with 70% rigidity in the tip and base using the RigiScan monitoring [165]. Upon discussing with his urologist, the patient began seeking penile prosthesis implant. After being explained and understanding the experimental nature of the proposed procedure, the patient signed informed consent. The procedure was approved by the relevant institutional review board. The patient was in otherwise good health. Tumor markers AFP, PSA, CA19-9, and CEA, hematology, biochemistry panel and coagulation were unremarkable. CT scans of the chest, ultrasound of the abdominal area, and fecal occult test were also unremarkable.

The patient was administered one tablet of Vocodin (7.5 mg hydrocodone) and one tablet of Xanax (1 mg) 30 minutes before the procedure. Local lidocaine was applied topically at the area of bone marrow puncture. A total of 60 ml of bone marrow aspirate was obtained and processed in a closed-system bone marrow concentration device. Bone marrow mononuclear cells were concentrated to a volume of 2 ml, with 1 ml administered into each cavernousal body using a 25 gauge syringe. A tourniquet was placed around the base of the penis during the injection procedure and held for 5 minutes to allow for maximal retention

No immediate injection-associated adverse events were noted. The patient reported a morning erection 2 days after cell administration. Although angiogenesis could not occur during this short time period, the possibility of bone marrow released nitric oxide stimulating erections via vasodilation may be postulated [166]. Three weeks after treatment, the patient reported erection strong enough for penetration, but did not have ability to sustain the erection until orgasm. At three month follow-up the patient reported having intercourse until orgasm several times and a marked increase in morning erections. Importantly, no adverse effects or ectopic tissue formation was observed at the 3, 12 and 18 month follow-up. At last visit, 18 months after procedure, the patient still reported improved sexual function as compared to prior to treatment.

## Conclusion

Bone marrow stem cell therapy has demonstrated therapeutic effects in clinical trials of heart failure and advanced peripheral artery disease. The rationale for bone marrow stem cell therapy of the penis in patients with erectile dysfunction is strong given that: a) The penile vasculature is the most endothelial-rich anatomical region of the body, thus even a small amount of therapeutic cells are likely to be incorporated; b) Blood flow in the flaccid penis is slower compared to systemic circulation, thus allowing for superior retention; and c) ease of injection given its external location. From an ethical perspective, the procedure of penile prosthesis implantation requires destruction of the cavernous, thus making it irreversible. The feasibility of the injection procedure, the fact that no adverse effects were noted, and the ease of the procedure, supports expanded clinical trials using this intervention.

## Competing interests

TW is the CEO and a shareholder of Creative Medical Health Inc.

## Authors’ contributions

TEI, TW, OC, JLC, and ANP reviewed the literature, wrote the paper, and proofread the final copy. All authors read and approved the final manuscript.

## References

[B1] Andersson KE Wagner G Physiology of penile erection Physiol Rev 1995 75 1 191 236 7831397 10.1152/physrev.1995.75.1.191

[B2] Fournier GR Jr Mechanisms of venous occlusion during canine penile erection: an anatomic demonstration J Urol 1987 137 1 163 167 3795360 10.1016/s0022-5347(17)43911-5

[B3] Toda N Ayajiki K Okamura T Nitric oxide and penile erectile function Pharmacol Ther 2005 106 2 233 266 10.1016/j.pharmthera.2004.11.011 15866322

[B4] Ravipati G Type 5 phosphodiesterase inhibitors in the treatment of erectile dysfunction and cardiovascular disease Cardiol Rev 2007 15 2 76 86 10.1097/01.crd.0000233904.77128.49 17303994

[B5] Dussault S Sildenafil increases endothelial progenitor cell function and improves ischemia-induced neovascularization in hypercholesterolemic apolipoprotein E-deficient mice Hypertension 2009 54 5 1043 1049 10.1161/HYPERTENSIONAHA.109.139451 19770400

[B6] Foresta C The PDE5 inhibitor sildenafil increases circulating endothelial progenitor cells and CXCR4 expression J Sex Med 2009 6 2 369 372 10.1111/j.1743-6109.2008.01014.x 18823318

[B7] Foresta C PDE-5 inhibitor, Vardenafil, increases circulating progenitor cells in humans Int J Impot Res 2005 17 4 377 380 10.1038/sj.ijir.3901325 15829988

[B8] Foresta C Relationship between vascular damage degrees and endothelial progenitor cells in patients with erectile dysfunction: effect of vardenafil administration and PDE5 expression in the bone marrow Eur Urol 2007 51 5 1411 1417 discussion 1417-9 10.1016/j.eururo.2006.08.052 17034932

[B9] Foresta C Effect of vardenafil on endothelial progenitor cells in hypogonadotrophic hypogonadal patients: role of testosterone treatment Clin Endocrinol 2009 71 3 412 416 10.1111/j.1365-2265.2008.03507.x 19094070

[B10] Ferrini MG Vardenafil prevents fibrosis and loss of corporal smooth muscle that occurs after bilateral cavernosal nerve resection in the rat Urology 2006 68 2 429 435 10.1016/j.urology.2006.05.011 16904479

[B11] Kovanecz I Long-term continuous sildenafil treatment ameliorates corporal veno-occlusive dysfunction (CVOD) induced by cavernosal nerve resection in rats Int J Impot Res 2008 20 2 202 212 10.1038/sj.ijir.3901612 17882231

[B12] Ozden E Effect of sildenafil citrate on penile weight and physiology of cavernous smooth muscle in a post-radical prostatectomy model of erectile dysfunction in rats Urology 2011 77 3 761 e1-7 21256544 10.1016/j.urology.2010.10.009

[B13] Padma-Nathan H McCullough A Forest C Erectile dysfunction secondary to nerve-sparing radical retropubic prostatectomy: comparative phosphodiesterase-5 inhibitor efficacy for therapy and novel prevention strategies Curr Urol Rep 2004 5 6 467 471 10.1007/s11934-004-0072-0 15541217

[B14] Sanna F Phosphodiesterase type 5 inhibitors facilitate noncontact erections in male rats: site of action in the brain and mechanism of action J Sex Med 2009 6 10 2680 2689 10.1111/j.1743-6109.2009.01410.x 19678884

[B15] Hatzimouratidis K Hatzichristou D Phosphodiesterase type 5 inhibitors: the day after Eur Urol 2007 51 1 75 88 discussion 89 10.1016/j.eururo.2006.07.020 16949200

[B16] Hatzimouratidis K Hatzichristou DG Phosphodiesterase type 5 inhibitors: unmet needs Curr Pharm Des 2009 15 30 3476 3485 10.2174/138161209789207042 19860693

[B17] Gentile V Preliminary observations on the use of propionyl-L-carnitine in combination with sildenafil in patients with erectile dysfunction and diabetes Curr Med Res Opin 2004 20 9 1377 1384 10.1185/030079904X2394 15383186

[B18] Morano S Antioxidant treatment associated with sildenafil reduces monocyte activation and markers of endothelial damage in patients with diabetic erectile dysfunction: a double-blind, placebo-controlled study Eur Urol 2007 52 6 1768 1774 10.1016/j.eururo.2007.04.042 17478034

[B19] Gutierrez P Hernandez P Mas M Combining programmed intracavernous PGE1 injections and sildenafil on demand to salvage sildenafil nonresponders Int J Impot Res 2005 17 4 354 358 10.1038/sj.ijir.3901290 15703770

[B20] Shabsigh R Randomized study of testosterone gel as adjunctive therapy to sildenafil in hypogonadal men with erectile dysfunction who do not respond to sildenafil alone J Urol 2008 179 5 Suppl S97 S102 18405769 10.1016/j.juro.2008.03.145

[B21] Greco EA Spera G Aversa A Combining testosterone and PDE5 inhibitors in erectile dysfunction: basic rationale and clinical evidences Eur Urol 2006 50 5 940 947 10.1016/j.eururo.2006.06.049 16979814

[B22] Chew KK Erectile dysfunction as a predictor for subsequent atherosclerotic cardiovascular events: findings from a linked-data study J Sex Med 2010 7 1 Pt 1 192 202 19912508 10.1111/j.1743-6109.2009.01576.x

[B23] Aversa A Endothelial dysfunction and erectile dysfunction in the aging man International journal of urology: official journal of the Japanese Urological Association 2010 17 1 38 47 10.1111/j.1442-2042.2009.02426.x 20002226

[B24] Chen SL Effect on left ventricular function of intracoronary transplantation of autologous bone marrow mesenchymal stem cell in patients with acute myocardial infarction Am J Cardiol 2004 94 1 92 95 10.1016/j.amjcard.2004.03.034 15219514

[B25] Chen SL Improvement of cardiac function after transplantation of autologous bone marrow mesenchymal stem cells in patients with acute myocardial infarction Chin Med J 2004 117 10 1443 1448 15498362

[B26] Katritsis DG Transcoronary transplantation of autologous mesenchymal stem cells and endothelial progenitors into infarcted human myocardium Catheterization and cardiovascular interventions: official journal of the Society for Cardiac Angiography & Interventions 2005 65 3 321 329 10.1002/ccd.20406 15954106

[B27] Chen S Intracoronary transplantation of autologous bone marrow mesenchymal stem cells for ischemic cardiomyopathy due to isolated chronic occluded left anterior descending artery J Invasive Cardiol 2006 18 11 552 556 17090821

[B28] Katritsis DG Electrophysiological effects of intracoronary transplantation of autologous mesenchymal and endothelial progenitor cells Europace: European pacing, arrhythmias, and cardiac electrophysiology: journal of the working groups on cardiac pacing, arrhythmias, and cardiac cellular electrophysiology of the European Society of Cardiology 2007 9 3 167 171 10.1093/europace/eul184 17272327

[B29] Mohyeddin-Bonab M Autologous in vitro expanded mesenchymal stem cell therapy for human old myocardial infarction Arch Iran Med 2007 10 4 467 473 17903051

[B30] Hare JM A randomized, double-blind, placebo-controlled, dose-escalation study of intravenous adult human mesenchymal stem cells (prochymal) after acute myocardial infarction J Am Coll Cardiol 2009 54 24 2277 2286 10.1016/j.jacc.2009.06.055 19958962 PMC3580848

[B31] Guhathakurta S Stem cell experiments and initial clinical trial of cellular cardiomyoplasty Asian Cardiovasc Thorac Ann 2009 17 6 581 586 10.1177/0218492309349363 20026532

[B32] Lasala GP Combination stem cell therapy for the treatment of medically refractory coronary ischemia: a Phase I study Cardiovascular revascularization medicine: including molecular interventions 2011 12 1 29 34 10.1016/j.carrev.2010.01.001 21241969

[B33] Lasala GP Combination stem cell therapy for the treatment of severe limb ischemia: safety and efficacy analysis Angiology 2010 61 6 551 556 10.1177/0003319710364213 20498146

[B34] Iwase T Comparison of angiogenic potency between mesenchymal stem cells and mononuclear cells in a rat model of hindlimb ischemia Cardiovasc Res 2005 66 3 543 551 10.1016/j.cardiores.2005.02.006 15914119

[B35] Lu D Comparison of bone marrow mesenchymal stem cells with bone marrow-derived mononuclear cells for treatment of diabetic critical limb ischemia and foot ulcer: A double-blind, randomized, controlled trial Diabetes Res Clin Pract 2011 92 1 26 36 10.1016/j.diabres.2010.12.010 21216483

[B36] Qiu X Combined Strategy of Mesenchymal Stem Cells Injection with VEGF Gene Therapy for the Treatment of Diabetes Associated Erectile Dysfunction J Androl 2011 33 1 37 44 21311050 10.2164/jandrol.110.012666

[B37] Qiu X Intracavernous transplantation of bone marrow-derived mesenchymal stem cells restores erectile function of streptozocin-induced diabetic rats J Sex Med 2011 8 2 427 436 10.1111/j.1743-6109.2010.02118.x 21091881

[B38] Kendirci M Transplantation of nonhematopoietic adult bone marrow stem/progenitor cells isolated by p75 nerve growth factor receptor into the penis rescues erectile function in a rat model of cavernous nerve injury J Urol 2010 184 4 1560 1566 10.1016/j.juro.2010.05.088 20728109 PMC3014289

[B39] Albersen M Injections of adipose tissue-derived stem cells and stem cell lysate improve recovery of erectile function in a rat model of cavernous nerve injury J Sex Med 2010 7 10 3331 3340 10.1111/j.1743-6109.2010.01875.x 20561166 PMC3885341

[B40] Abdel Aziz MT Effect of mesenchymal stem cell penile transplantation on erectile signaling of aged rats Andrologia 2010 42 3 187 192 10.1111/j.1439-0272.2009.00977.x 20500748

[B41] Huang YC The effect of intracavernous injection of adipose tissue-derived stem cells on hyperlipidemia-associated erectile dysfunction in a rat model J Sex Med 2010 7 4 Pt 1 1391 1400 20141586 10.1111/j.1743-6109.2009.01697.x PMC3163604

[B42] Garcia MM Treatment of erectile dysfunction in the obese type 2 diabetic ZDF rat with adipose tissue-derived stem cells J Sex Med 2010 7 1 Pt 1 89 98 20104670 10.1111/j.1743-6109.2009.01541.x PMC2904063

[B43] Lin G Potential of adipose-derived stem cells for treatment of erectile dysfunction J Sex Med 2009 6 Suppl 3 320 327 19267855 10.1111/j.1743-6109.2008.01190.x PMC2895916

[B44] Nolazco G Effect of muscle-derived stem cells on the restoration of corpora cavernosa smooth muscle and erectile function in the aged rat BJU Int 2008 101 9 1156 1164 10.1111/j.1464-410X.2008.07507.x 18294308

[B45] Song YS Magnetic resonance evaluation of human mesenchymal stem cells in corpus cavernosa of rats and rabbits Asian J Androl 2007 9 3 361 367 10.1111/j.1745-7262.2007.00265.x 17486277

[B46] Song YS Potential differentiation of human mesenchymal stem cell transplanted in rat corpus cavernosum toward endothelial or smooth muscle cells Int J Impot Res 2007 19 4 378 385 10.1038/sj.ijir.3901539 17460699

[B47] Bivalacqua TJ Mesenchymal stem cells alone or ex vivo gene modified with endothelial nitric oxide synthase reverse age-associated erectile dysfunction Am J Physiol Heart Circ Physiol 2007 292 3 H1278 H1290 17071732 10.1152/ajpheart.00685.2006

[B48] Bochinski D The effect of neural embryonic stem cell therapy in a rat model of cavernosal nerve injury BJU Int 2004 94 6 904 909 10.1111/j.1464-410X.2003.05057.x 15476533

[B49] Wespes E Schulman CC Erectile dysfunction and cardiovascular diseases Arch Esp Urol 2010 63 8 649 654 21045247

[B50] Asahara T Isolation of putative progenitor endothelial cells for angiogenesis Science 1997 275 5302 964 967 10.1126/science.275.5302.964 9020076

[B51] Sieveking DP Strikingly different angiogenic properties of endothelial progenitor cell subpopulations: insights from a novel human angiogenesis assay J Am Coll Cardiol 2008 51 6 660 668 10.1016/j.jacc.2007.09.059 18261686

[B52] Peichev M Expression of VEGFR-2 and AC133 by circulating human CD34(+) cells identifies a population of functional endothelial precursors Blood 2000 95 3 952 958 10648408

[B53] Wojakowski W Tendera M Mobilization of bone marrow-derived progenitor cells in acute coronary syndromes Folia histochemica et cytobiologica / Polish Academy of Sciences, Polish Histochemical and Cytochemical Society 2005 43 4 229 232 16382890

[B54] Massa M Increased circulating hematopoietic and endothelial progenitor cells in the early phase of acute myocardial infarction Blood 2005 105 1 199 206 10.1182/blood-2004-05-1831 15345590

[B55] Voo S Enhanced functional response of CD133+ circulating progenitor cells in patients early after acute myocardial infarction Eur Heart J 2008 29 2 241 250 18156140 10.1093/eurheartj/ehm542

[B56] Bogoslovsky T Endothelial progenitor cells correlate with lesion volume and growth in acute stroke Neurology 2010 75 23 2059 2062 10.1212/WNL.0b013e318200d741 21135380 PMC2995536

[B57] Navarro-Sobrino M Mobilization, endothelial differentiation and functional capacity of endothelial progenitor cells after ischemic stroke Microvasc Res 2010 80 3 317 323 10.1016/j.mvr.2010.05.008 20594997

[B58] Bogoslovsky T Stromal-derived factor-1[alpha] correlates with circulating endothelial progenitor cells and with acute lesion volume in stroke patients Stroke; a journal of cerebral circulation 2011 42 3 618 625 10.1161/STROKEAHA.110.596007 21257825 PMC4751042

[B59] Sobrino T The increase of circulating endothelial progenitor cells after acute ischemic stroke is associated with good outcome Stroke; a journal of cerebral circulation 2007 38 10 2759 2764 10.1161/STROKEAHA.107.484386 17761925

[B60] Schmidt-Lucke C Reduced number of circulating endothelial progenitor cells predicts future cardiovascular events: proof of concept for the clinical importance of endogenous vascular repair Circulation 2005 111 22 2981 2987 10.1161/CIRCULATIONAHA.104.504340 15927972

[B61] Kupatt C Retroinfusion of embryonic endothelial progenitor cells attenuates ischemia-reperfusion injury in pigs: role of phosphatidylinositol 3-kinase/AKT kinase Circulation 2005 112 9 Suppl I117 I122 16159802 10.1161/CIRCULATIONAHA.104.524801

[B62] Aicher A Assessment of the tissue distribution of transplanted human endothelial progenitor cells by radioactive labeling Circulation 2003 107 16 2134 2139 10.1161/01.CIR.0000062649.63838.C9 12695305

[B63] Fan Y Endothelial progenitor cell transplantation improves long-term stroke outcome in mice Ann Neurol 2010 67 4 488 497 10.1002/ana.21919 20437584 PMC3026588

[B64] Lam CF Transplantation of endothelial progenitor cells improves pulmonary endothelial function and gas exchange in rabbits with endotoxin-induced acute lung injury Anesth Analg 2011 112 3 620 627 10.1213/ANE.0b013e3182075da4 21233499

[B65] Mao M Intravenous delivery of bone marrow-derived endothelial progenitor cells improves survival and attenuates lipopolysaccharide-induced lung injury in rats Shock 2010 34 2 196 204 10.1097/SHK.0b013e3181d49457 20090567

[B66] Kahler CM Peripheral infusion of rat bone marrow derived endothelial progenitor cells leads to homing in acute lung injury Respir Res 2007 8 50 10.1186/1465-9921-8-50 17620112 PMC2000890

[B67] Elkhafif N CD133(+) human umbilical cord blood stem cells enhance angiogenesis in experimental chronic hepatic fibrosis APMIS: acta pathologica, microbiologica, et immunologica Scandinavica 2011 119 1 66 75 10.1111/j.1600-0463.2010.02693.x 21143528

[B68] Liu F Transplanted endothelial progenitor cells ameliorate carbon tetrachloride-induced liver cirrhosis in rats Liver transplantation: official publication of the American Association for the Study of Liver Diseases and the International Liver Transplantation Society 2009 15 9 1092 1100 10.1002/lt.21845 19718641

[B69] Nakamura T Significance and therapeutic potential of endothelial progenitor cell transplantation in a cirrhotic liver rat model Gastroenterology 2007 133 1 91 107 e1 10.1053/j.gastro.2007.03.110 17631135

[B70] Wang SH Late outgrowth endothelial cells derived from Wharton jelly in human umbilical cord reduce neointimal formation after vascular injury: involvement of pigment epithelium-derived factor Arterioscler Thromb Vasc Biol 2009 29 6 816 822 10.1161/ATVBAHA.109.184739 19342598

[B71] Desouza CV Role of inflammation and insulin resistance in endothelial progenitor cell dysfunction Diabetes 2011 60 4 1286 1294 10.2337/db10-0875 21346178 PMC3064102

[B72] Orlic D Mobilized bone marrow cells repair the infarcted heart, improving function and survival Proc Natl Acad Sci USA 2001 98 18 10344 10349 10.1073/pnas.181177898 11504914 PMC56963

[B73] Zhang L Granulocyte colony-stimulating factor treatment ameliorates liver injury and improves survival in rats with d-galactosamine-induced acute liver failure Toxicol Lett 2011 204 1 92 99 10.1016/j.toxlet.2011.04.016 21550386

[B74] Cho HJ Mobilized endothelial progenitor cells by granulocyte-macrophage colony-stimulating factor accelerate reendothelialization and reduce vascular inflammation after intravascular radiation Circulation 2003 108 23 2918 2925 10.1161/01.CIR.0000097001.79750.78 14568896

[B75] Lara-Hernandez R Safety and efficacy of therapeutic angiogenesis as a novel treatment in patients with critical limb ischemia Ann Vasc Surg 2010 24 2 287 294 10.1016/j.avsg.2009.10.012 20142004

[B76] Kim DI Angiogenesis facilitated by autologous whole bone marrow stem cell transplantation for Buerger’s disease Stem Cells 2006 24 5 1194 1200 10.1634/stemcells.2005-0349 16439614

[B77] Mund JA Endothelial progenitor cells and cardiovascular cell-based therapies Cytotherapy 2009 11 2 103 113 10.1080/14653240802714827 19241233

[B78] Franck-Lissbrant I Testosterone stimulates angiogenesis and vascular regrowth in the ventral prostate in castrated adult rats Endocrinology 1998 139 2 451 456 10.1210/en.139.2.451 9449610

[B79] Sieveking DP Chow RW Ng MK Androgens, angiogenesis and cardiovascular regeneration Curr Opin Endocrinol Diabetes Obes 2010 17 3 277 283 10.1097/MED.0b013e3283394e20 20389240

[B80] Sieveking DP A sex-specific role for androgens in angiogenesis J Exp Med 2010 207 2 345 352 10.1084/jem.20091924 20071503 PMC2822613

[B81] Liu PY Death AK Handelsman DJ Androgens and cardiovascular disease Endocr Rev 2003 24 3 313 340 12788802 10.1210/er.2003-0005

[B82] Yiu YF Vitamin d deficiency is associated with depletion of circulating endothelial progenitor cells and endothelial dysfunction in patients with type 2 diabetes J Clin Endocrinol Metabol 2011 96 5 E830 E835 10.1210/jc.2010-2212 21325459

[B83] Kahn MB Insulin resistance impairs circulating angiogenic progenitor cell function and delays endothelial regeneration Diabetes 2011 60 4 1295 1303 10.2337/db10-1080 21317296 PMC3064103

[B84] Li M The decrement in circulating endothelial progenitor cells (EPCs) in type 2 diabetes is independent of the severity of the hypoadiponectemia Diabetes Metab Res Rev 2011 27 2 185 194 10.1002/dmrr.1159 21294240

[B85] Mieno S Effects of diabetes mellitus on VEGF-induced proliferation response in bone marrow derived endothelial progenitor cells J Card Surg 2010 25 5 618 625 10.1111/j.1540-8191.2010.01086.x 20626511 PMC2958227

[B86] Reinhard H Multifactorial treatment increases endothelial progenitor cells in patients with type 2 diabetes Diabetologia 2010 53 10 2129 2133 10.1007/s00125-010-1843-4 20607514

[B87] Ramunni A Effect of low-density lipoprotein apheresis on circulating endothelial progenitor cells in familial hypercholesterolemia Blood Purif 2010 29 4 383 389 10.1159/000314650 20484899

[B88] Tie G Oxidized low-density lipoprotein induces apoptosis in endothelial progenitor cells by inactivating the phosphoinositide 3-kinase/Akt pathway J Vasc Res 2010 47 6 519 530 10.1159/000313879 20431300 PMC2945270

[B89] Rossi F HDL cholesterol is a strong determinant of endothelial progenitor cells in hypercholesterolemic subjects Microvasc Res 2010 80 2 274 279 10.1016/j.mvr.2010.05.003 20478316

[B90] Pirro M Hypercholesterolemia-associated endothelial progenitor cell dysfunction Ther Adv Cardiovasc Dis 2008 2 5 329 339 10.1177/1753944708094769 19124431

[B91] Cheng J Oxidized low-density lipoprotein stimulates p53-dependent activation of proapoptotic Bax leading to apoptosis of differentiated endothelial progenitor cells Endocrinology 2007 148 5 2085 2094 10.1210/en.2006-1709 17289842

[B92] Croce G Nonpharmacological treatment of hypercholesterolemia increases circulating endothelial progenitor cell population in adults Arterioscler Thromb Vasc Biol 2006 26 5 e38 e39 10.1161/01.ATV.0000218504.71680.b5 16627813

[B93] Heida NM Effects of obesity and weight loss on the functional properties of early outgrowth endothelial progenitor cells J Am Coll Cardiol 2010 55 4 357 367 10.1016/j.jacc.2009.09.031 20117442

[B94] Tobler K Reduction of both number and proliferative activity of human endothelial progenitor cells in obesity Int J Obes 2010 34 4 687 700 10.1038/ijo.2009.280 20065973

[B95] Zenovich AG Taylor DA Atherosclerosis as a disease of failed endogenous repair Frontiers in bioscience: a journal and virtual library 2008 13 3621 3636 18508460 10.2741/2954 PMC2666301

[B96] Tousoulis D Role of inflammation and oxidative stress in endothelial progenitor cell function and mobilization: therapeutic implications for cardiovascular diseases Atherosclerosis 2008 201 2 236 247 10.1016/j.atherosclerosis.2008.05.034 18599065

[B97] Kondo T Smoking cessation rapidly increases circulating progenitor cells in peripheral blood in chronic smokers Arterioscler Thromb Vasc Biol 2004 24 8 1442 1447 10.1161/01.ATV.0000135655.52088.c5 15191940

[B98] Baumhakel M Circulating endothelial progenitor cells correlate with erectile function in patients with coronary heart disease Eur Heart J 2006 27 18 2184 2188 10.1093/eurheartj/ehl202 16926179

[B99] Esposito K Circulating CD34+ KDR+ endothelial progenitor cells correlate with erectile function and endothelial function in overweight men J Sex Med 2009 6 1 107 114 10.1111/j.1743-6109.2008.01042.x 19170841

[B100] Foresta C Increased levels of osteocalcin-positive endothelial progenitor cells in patients affected by erectile dysfunction and cavernous atherosclerosis J Sex Med 2010 7 2 Pt 1 751 757 19796016 10.1111/j.1743-6109.2009.01520.x

[B101] Shao H Statin and stromal cell-derived factor-1 additively promote angiogenesis by enhancement of progenitor cells incorporation into new vessels Stem Cells 2008 26 5 1376 1384 10.1634/stemcells.2007-0785 18308946

[B102] Zaruba MM Franz WM Role of the SDF-1-CXCR4 axis in stem cell-based therapies for ischemic cardiomyopathy Expert Opin Biol Ther 2010 10 3 321 335 10.1517/14712590903460286 20132055

[B103] Hiasa K Gene transfer of stromal cell-derived factor-1alpha enhances ischemic vasculogenesis and angiogenesis via vascular endothelial growth factor/endothelial nitric oxide synthase-related pathway: next-generation chemokine therapy for therapeutic neovascularization Circulation 2004 109 20 2454 2461 10.1161/01.CIR.0000128213.96779.61 15148275

[B104] Ryu JK Downregulation of angiogenic factors and their downstream target molecules affects the deterioration of erectile function in a rat model of hypercholesterolemia Urology 2006 67 6 1329 1334 10.1016/j.urology.2005.12.027 16750245

[B105] Xie D A VEGF trap inhibits the beneficial effect of bFGF on vasoreactivity in corporal tissues of hypercholesterolemic rabbits J Sex Med 2008 5 9 2069 2078 10.1111/j.1743-6109.2008.00933.x 18637998

[B106] Xie D Decreases in corporeal vascular endothelial growth factor expression precede vasoreactivity changes in cholesterol fed rabbits J Urol 2005 173 4 1418 1422 10.1097/01.ju.0000149035.98638.33 15758816

[B107] De Young L Arteriogenic erectile dysfunction alters protein expression within the cavernosal tissue in an animal model J Sex Med 2005 2 2 199 206 10.1111/j.1743-6109.2005.20229.x 16422887

[B108] Rajasekaran M Altered growth factor expression in the aging penis: the Brown-Norway rat model J Androl 2002 23 3 393 399 12002441

[B109] Chang Hwang E Effects of Androgen on the Expression of Vascular Endothelial Growth Factor in the Penile Corpus Cavernosum Urology 2011 77 6 1381 1386 10.1016/j.urology.2011.01.036 21458034

[B110] Wang T Alterations in angiogenic growth factors and neuronal nitric oxide synthase expression in chronic cavernosal ischemia Int J Impot Res 2004 16 5 403 411 10.1038/sj.ijir.3901186 14999219

[B111] Byrne RR Vascular endothelial growth factor restores corporeal smooth muscle function in vitro J Urol 2001 165 4 1310 1315 10.1016/S0022-5347(01)69890-2 11257707

[B112] Henry GD Intracavernosal injections of vascular endothelial growth factor protects endothelial dependent corpora cavernosal smooth muscle relaxation in the hypercholesterolemic rabbit: a preliminary study Int J Impot Res 2000 12 6 334 339 10.1038/sj.ijir.3900621 11416837

[B113] Rogers RS Intracavernosal vascular endothelial growth factor (VEGF) injection and adeno-associated virus-mediated VEGF gene therapy prevent and reverse venogenic erectile dysfunction in rats Int J Impot Res 2003 15 1 26 37 10.1038/sj.ijir.3900943 12605238

[B114] Park K Intracavernosal injection of vascular endothelial growth factor improves erectile function in aged rats Eur Urol 2004 46 3 403 407 10.1016/j.eururo.2004.04.032 15306114

[B115] Dall’Era JE Vascular endothelial growth factor (VEGF) gene therapy using a nonviral gene delivery system improves erectile function in a diabetic rat model Int J Impot Res 2008 20 3 307 314 10.1038/ijir.2008.1 18273028

[B116] Hsieh PS The effect of vascular endothelial growth factor and brain-derived neurotrophic factor on cavernosal nerve regeneration in a nerve-crush rat model BJU Int 2003 92 4 470 475 10.1046/j.1464-410X.2003.04373.x 12930443

[B117] Lin CS Intracavernosal injection of vascular endothelial growth factor induces nitric oxide synthase isoforms BJU Int 2002 89 9 955 960 10.1046/j.1464-410X.2002.02792.x 12010247

[B118] Musicki B Phosphorylated endothelial nitric oxide synthase mediates vascular endothelial growth factor-induced penile erection Biol Reprod 2004 70 2 282 289 10.1095/biolreprod.103.021113 14522830

[B119] Yamanaka M Vascular endothelial growth factor restores erectile function through inhibition of apoptosis in diabetic rat penile crura J Urol 2005 173 1 318 323 10.1097/01.ju.0000141586.46822.44 15592104

[B120] Shirai M Vascular endothelial growth factor restores erectile function through modulation of the insulin-like growth factor system and sex hormone receptors in diabetic rat Biochem Biophys Res Commun 2006 341 3 755 762 10.1016/j.bbrc.2005.12.226 16455052

[B121] Dai Q Systemic basic fibroblast growth factor induces favorable histological changes in the corpus cavernosum of hypercholesterolemic rabbits J Urol 2003 170 2 Pt 1 664 668 12853852 10.1097/01.ju.0000065247.55066.ad

[B122] Xie D Intracavernosal basic fibroblast growth factor improves vasoreactivity in the hypercholesterolemic rabbit J Sex Med 2006 3 2 223 232 10.1111/j.1743-6109.2005.00174.x 16490015

[B123] Rabinovsky ED The multifunctional role of IGF-1 in peripheral nerve regeneration Neurol Res 2004 26 2 204 210 10.1179/016164104225013851 15072640

[B124] Abdel-Gawad M Huynh H Brock GB Experimental chronic renal failure-associated erectile dysfunction: molecular alterations in nitric oxide synthase pathway and IGF-I system Mol Urol 1999 3 2 117 125 10851313

[B125] Schaaf L Stalla GK [Endocrine changes with advancing age] MMW Fortschr Med 2009 151 42 43 45 19938780

[B126] Probst-Hensch NM Chronic age-related diseases share risk factors: do they share pathophysiological mechanisms and why does that matter? Swiss Med Wkly 2010 140 w13072 20809438 10.4414/smw.2010.13072

[B127] Jung GW IGF-I and TGF-beta2 have a key role on regeneration of nitric oxide synthase (NOS)-containing nerves after cavernous neurotomy in rats Int J Impot Res 1999 11 5 247 259 10.1038/sj.ijir.3900402 10553803

[B128] Pu XY Improvement in erectile dysfunction after insulin-like growth factor-1 gene therapy in diabetic rats Asian J Androl 2007 9 1 83 91 10.1111/j.1745-7262.2007.00215.x 16855763

[B129] Pu XY Insulin-like growth factor-1 restores erectile function in aged rats: modulation the integrity of smooth muscle and nitric oxide-cyclic guanosine monophosphate signaling activity J Sex Med 2008 5 6 1345 1354 10.1111/j.1743-6109.2008.00817.x 18355170

[B130] Hinkel R Trenkwalder T Kupatt C Gene therapy for ischemic heart disease Expert Opin Biol Ther 2011 11 6 723 737 10.1517/14712598.2011.570749 21434842

[B131] Melman A hMaxi-K gene transfer in males with erectile dysfunction: results of the first human trial Hum Gene Ther 2006 17 12 1165 1176 10.1089/hum.2006.17.1165 17134370

[B132] Ichim TE Combination stem cell therapy for heart failure International archives of medicine 2010 3 1 5 10.1186/1755-7682-3-5 20398245 PMC3003238

[B133] Yu XY The effects of mesenchymal stem cells on c-kit up-regulation and cell-cycle re-entry of neonatal cardiomyocytes are mediated by activation of insulin-like growth factor 1 receptor Mol Cell Biochem 2009 332 1–2 25 32 19507001 10.1007/s11010-009-0170-x

[B134] Shabbir A Heart failure therapy mediated by the trophic activities of bone marrow mesenchymal stem cells: a noninvasive therapeutic regimen Am J Physiol Heart Circ Physiol 2009 296 6 H1888 H1897 10.1152/ajpheart.00186.2009 19395555 PMC2716100

[B135] Chen L Paracrine factors of mesenchymal stem cells recruit macrophages and endothelial lineage cells and enhance wound healing PLoS One 2008 3 4 e1886 10.1371/journal.pone.0001886 18382669 PMC2270908

[B136] Fan W Crawford R Xiao Y The ratio of VEGF/PEDF expression in bone marrow mesenchymal stem cells regulates neovascularization Differentiation; research in biological diversity 2011 81 3 181 191 10.1016/j.diff.2010.12.003 21236558

[B137] Jiang SY Xie XT Zhou JJ [Gene profile and fibroblast growth factor 2 expression in mesenchymal stem cell from children with aplastic anemia] Zhonghua er ke za zhi. Chinese journal of pediatrics 2009 47 3 218 220 19573438

[B138] Ichim TE Mesenchymal stem cells as anti-inflammatories: implications for treatment of Duchenne muscular dystrophy Cell Immunol 2010 260 2 75 82 10.1016/j.cellimm.2009.10.006 19917503

[B139] Bae KS Neuron-like differentiation of bone marrow-derived mesenchymal stem cells Yonsei Med J 2011 52 3 401 412 10.3349/ymj.2011.52.3.401 21488182 PMC3101055

[B140] Riordan NH Non-expanded adipose stromal vascular fraction cell therapy for multiple sclerosis J Transl Med 2009 7 29 10.1186/1479-5876-7-29 19393041 PMC2679713

[B141] Zhong Z Feasibility investigation of allogeneic endometrial regenerative cells J Transl Med 2009 7 15 10.1186/1479-5876-7-15 19232091 PMC2649897

[B142] Bahk JY Treatment of diabetic impotence with umbilical cord blood stem cell intracavernosal transplant: preliminary report of 7 cases Experimental and clinical transplantation: official journal of the Middle East Society for Organ Transplantation 2010 8 2 150 160 20565373

[B143] Thomas ED Supralethal whole body irradiation and isologous marrow transplantation in man J Clin Invest 1959 38 1709 1716 10.1172/JCI103949 13837954 PMC444138

[B144] Kocher AA Neovascularization of ischemic myocardium by human bone-marrow-derived angioblasts prevents cardiomyocyte apoptosis, reduces remodeling and improves cardiac function Nat Med 2001 7 4 430 436 10.1038/86498 11283669

[B145] Fazel S Cardioprotective c-kit+ cells are from the bone marrow and regulate the myocardial balance of angiogenic cytokines J Clin Invest 2006 116 7 1865 1877 10.1172/JCI27019 16823487 PMC1483161

[B146] Orlic D Adult bone marrow stem cells regenerate myocardium in ischemic heart disease Ann N Y Acad Sci 2003 996 152 157 10.1111/j.1749-6632.2003.tb03243.x 12799293

[B147] Strauer BE [Intracoronary, human autologous stem cell transplantation for myocardial regeneration following myocardial infarction] Deutsche medizinische Wochenschrift 2001 126 34-35 932 938 11523014 10.1055/s-2001-16579-2

[B148] Assmus B Transplantation of progenitor cells and regeneration enhancement in acute myocardial infarction (TOPCARE-AMI) Circulation 2002 106 24 3009 3017 10.1161/01.CIR.0000043246.74879.CD 12473544

[B149] Yousef M The BALANCE Study: clinical benefit and long-term outcome after intracoronary autologous bone marrow cell transplantation in patients with acute myocardial infarction J Am Coll Cardiol 2009 53 24 2262 2269 10.1016/j.jacc.2009.02.051 19520249

[B150] Meyer GP Intracoronary bone marrow cell transfer after myocardial infarction: 5-year follow-up from the randomized-controlled BOOST trial Eur Heart J 2009 30 24 2978 2984 10.1093/eurheartj/ehp374 19773226

[B151] Schachinger V Transplantation of progenitor cells and regeneration enhancement in acute myocardial infarction: final one-year results of the TOPCARE-AMI Trial J Am Coll Cardiol 2004 44 8 1690 1699 10.1016/j.jacc.2004.08.014 15489105

[B152] Wen Y Direct autologous bone marrow-derived stem cell transplantation for ischemic heart disease: a meta-analysis Expert Opin Biol Ther 2011 11 5 559 567 10.1517/14712598.2011.560567 21388335

[B153] Zhang C Efficacy and safety of intracoronary autologous bone marrow-derived cell transplantation in patients with acute myocardial infarction: insights from randomized controlled trials with 12 or more months follow-up Clin Cardiol 2010 33 6 353 360 10.1002/clc.20745 20556805 PMC6653268

[B154] Hamano K Local implantation of autologous bone marrow cells for therapeutic angiogenesis in patients with ischemic heart disease: clinical trial and preliminary results Jpn Circ J 2001 65 9 845 847 10.1253/jcj.65.845 11548889

[B155] Beeres SL Usefulness of intramyocardial injection of autologous bone marrow-derived mononuclear cells in patients with severe angina pectoris and stress-induced myocardial ischemia Am J Cardiol 2006 97 9 1326 1331 10.1016/j.amjcard.2005.11.068 16635605

[B156] van Ramshorst J Intramyocardial bone marrow cell injection for chronic myocardial ischemia: a randomized controlled trial JAMA 2009 301 19 1997 2004 10.1001/jama.2009.685 19454638

[B157] Ziegler MA Marvels, mysteries, and misconceptions of vascular compensation to peripheral artery occlusion Microcirculation 2010 17 1 3 20 10.1111/j.1549-8719.2010.00008.x 20141596 PMC2909670

[B158] Tateishi-Yuyama E Therapeutic angiogenesis for patients with limb ischaemia by autologous transplantation of bone-marrow cells: a pilot study and a randomised controlled trial Lancet 2002 360 9331 427 435 10.1016/S0140-6736(02)09670-8 12241713

[B159] Nizankowski R The treatment of advanced chronic lower limb ischaemia with marrow stem cell autotransplantation Kardiol Pol 2005 63 4 351 360 discussion 361 16273471

[B160] Hernandez P Autologous bone-marrow mononuclear cell implantation in patients with severe lower limb ischaemia: a comparison of using blood cell separator and Ficoll density gradient centrifugation Atherosclerosis 2007 194 2 e52 e56 10.1016/j.atherosclerosis.2006.08.025 16982058

[B161] Amann B Autologous bone marrow cell transplantation increases leg perfusion and reduces amputations in patients with advanced critical limb ischemia due to peripheral artery disease Cell Transplant 2009 18 3 371 380 10.3727/096368909788534942 19500466

[B162] Couto BG Bone marrow mononuclear cell therapy for patients with cirrhosis: a Phase 1 study Liver international: official journal of the International Association for the Study of the Liver 2011 31 3 391 400 10.1111/j.1478-3231.2010.02424.x 21281433

[B163] Suarez-Monteagudo C Autologous bone marrow stem cell neurotransplantation in stroke patients An open study. Restorative neurology and neuroscience 2009 27 3 151 161 10.3233/RNN-2009-0483 19531871

[B164] Taguchi A Administration of CD34+ cells after stroke enhances neurogenesis via angiogenesis in a mouse model J Clin Invest 2004 114 3 330 338 15286799 10.1172/JCI20622 PMC484977

[B165] Elhanbly S Erectile dysfunction in smokers: a penile dynamic and vascular study J Androl 2004 25 6 991 995 15477374 10.1002/j.1939-4640.2004.tb03172.x

[B166] You D Increase in vascular permeability and vasodilation are critical for proangiogenic effects of stem cell therapy Circulation 2006 114 4 328 338 10.1161/CIRCULATIONAHA.105.589937 16847153

